# Evaluating the role of anxiety on the association between irritable bowel syndrome and brain volumes: a mediation analysis in the UK Biobank cohort

**DOI:** 10.1093/braincomms/fcad116

**Published:** 2023-04-11

**Authors:** Peilin Meng, Bolun Cheng, Chuyu Pan, Li Liu, Shiqiang Cheng, Xuena Yang, Yujing Chen, Chun’e Li, Huijie Zhang, Zhen Zhang, Jingxi Zhang, Dan He, Sirong Shi, Xiaoge Chu, Qingqing Cai, Na Zhang, Xiaoyue Qin, Yijing Zhao, Wenming Wei, Yumeng Jia, Yan Wen, Feng Zhang

**Affiliations:** Key Laboratory of Trace Elements and Endemic Diseases of National Health and Family Planning Commission, School of Public Health, Health Science Center, Xi’an Jiaotong University, Xi’an 710061, China; Key Laboratory of Trace Elements and Endemic Diseases of National Health and Family Planning Commission, School of Public Health, Health Science Center, Xi’an Jiaotong University, Xi’an 710061, China; Key Laboratory of Trace Elements and Endemic Diseases of National Health and Family Planning Commission, School of Public Health, Health Science Center, Xi’an Jiaotong University, Xi’an 710061, China; Key Laboratory of Trace Elements and Endemic Diseases of National Health and Family Planning Commission, School of Public Health, Health Science Center, Xi’an Jiaotong University, Xi’an 710061, China; Key Laboratory of Trace Elements and Endemic Diseases of National Health and Family Planning Commission, School of Public Health, Health Science Center, Xi’an Jiaotong University, Xi’an 710061, China; Key Laboratory of Trace Elements and Endemic Diseases of National Health and Family Planning Commission, School of Public Health, Health Science Center, Xi’an Jiaotong University, Xi’an 710061, China; Key Laboratory of Trace Elements and Endemic Diseases of National Health and Family Planning Commission, School of Public Health, Health Science Center, Xi’an Jiaotong University, Xi’an 710061, China; Key Laboratory of Trace Elements and Endemic Diseases of National Health and Family Planning Commission, School of Public Health, Health Science Center, Xi’an Jiaotong University, Xi’an 710061, China; Key Laboratory of Trace Elements and Endemic Diseases of National Health and Family Planning Commission, School of Public Health, Health Science Center, Xi’an Jiaotong University, Xi’an 710061, China; Key Laboratory of Trace Elements and Endemic Diseases of National Health and Family Planning Commission, School of Public Health, Health Science Center, Xi’an Jiaotong University, Xi’an 710061, China; Key Laboratory of Trace Elements and Endemic Diseases of National Health and Family Planning Commission, School of Public Health, Health Science Center, Xi’an Jiaotong University, Xi’an 710061, China; Key Laboratory of Trace Elements and Endemic Diseases of National Health and Family Planning Commission, School of Public Health, Health Science Center, Xi’an Jiaotong University, Xi’an 710061, China; Key Laboratory of Trace Elements and Endemic Diseases of National Health and Family Planning Commission, School of Public Health, Health Science Center, Xi’an Jiaotong University, Xi’an 710061, China; Key Laboratory of Trace Elements and Endemic Diseases of National Health and Family Planning Commission, School of Public Health, Health Science Center, Xi’an Jiaotong University, Xi’an 710061, China; Key Laboratory of Trace Elements and Endemic Diseases of National Health and Family Planning Commission, School of Public Health, Health Science Center, Xi’an Jiaotong University, Xi’an 710061, China; Key Laboratory of Trace Elements and Endemic Diseases of National Health and Family Planning Commission, School of Public Health, Health Science Center, Xi’an Jiaotong University, Xi’an 710061, China; Key Laboratory of Trace Elements and Endemic Diseases of National Health and Family Planning Commission, School of Public Health, Health Science Center, Xi’an Jiaotong University, Xi’an 710061, China; Key Laboratory of Trace Elements and Endemic Diseases of National Health and Family Planning Commission, School of Public Health, Health Science Center, Xi’an Jiaotong University, Xi’an 710061, China; Key Laboratory of Trace Elements and Endemic Diseases of National Health and Family Planning Commission, School of Public Health, Health Science Center, Xi’an Jiaotong University, Xi’an 710061, China; Key Laboratory of Trace Elements and Endemic Diseases of National Health and Family Planning Commission, School of Public Health, Health Science Center, Xi’an Jiaotong University, Xi’an 710061, China; Key Laboratory of Trace Elements and Endemic Diseases of National Health and Family Planning Commission, School of Public Health, Health Science Center, Xi’an Jiaotong University, Xi’an 710061, China; Key Laboratory of Trace Elements and Endemic Diseases of National Health and Family Planning Commission, School of Public Health, Health Science Center, Xi’an Jiaotong University, Xi’an 710061, China

**Keywords:** anxiety, irritable bowel syndrome, brain volumes, mediation analysis

## Abstract

There is a strong link between irritable bowel syndrome and brain volumes, yet, to date, research examining the mediators of this association has been little. Based on the phenotypic data of 15 248 participants from the UK Biobank, a two-stage mediation analysis was performed to assess the association among brain volumes, anxiety, and irritable bowel syndrome. In the first stage, we identified the candidate mediating role of anxiety for irritable bowel syndrome associated with brain volumes using regression models. Then, we quantified the magnitude of the mediation effects by evaluating the average causal-mediated effect and proportion of mediation through performing mediation analyses in the R package in the second stage. In the first stage, we identified the partly mediating role of anxiety in the association between irritable bowel syndrome and the volume of thalamus (*P*_left_ = 1.16 × 10^−4^, *P*_right_ = 2.41 × 10^−4^), and grey matter (*P*_left_ = 3.22 × 10^−2^, *P*_right_ = 1.18 × 10^−2^) in the VIIIa cerebellum. In the second stage, we observed that the proportion of the total effect of irritable bowel syndrome on volume of thalamus mediated by anxiety was 14.3% for the left region (*β*_Average causal-mediated effect_ = −0.008, *P*_Average causal-mediated effect_ = 0.004) and 14.6% for the right region (*β*_Average causal-mediated effect_ = −0.007, *P*_Average causal-mediated effect_ = 0.006). Anxiety mediated 30.8% for the left region (*β*_Average causal-mediated effect_ = −0.013, *P*_Average causal-mediated effect_ = 0.002) and 21.6% for the right region (*β*_Average causal-mediated effect_ = −0.010, *P*_Average causal-mediated effect_ x= 0.018) of the total effect of irritable bowel syndrome on the volume of grey matter in the VIIIa cerebellum. Our study revealed the indirect mediating role of anxiety in the association between irritable bowel syndrome and brain volumes, promoting our understanding of the functional mechanisms of irritable bowel syndrome and its related psychosocial factors.

## Teaser

Meng *et al*. conducted mediation analyses to identify the partly mediating role of anxiety in the association between irritable bowel syndrome and volume of thalamus and volume of grey matter in the VIIIa cerebellum and then detect their mediation proportion, promoting our understanding of the mechanisms of irritable bowel syndrome and related psychosocial factors.

## Graphical Abstract

**Graphical abstract:**
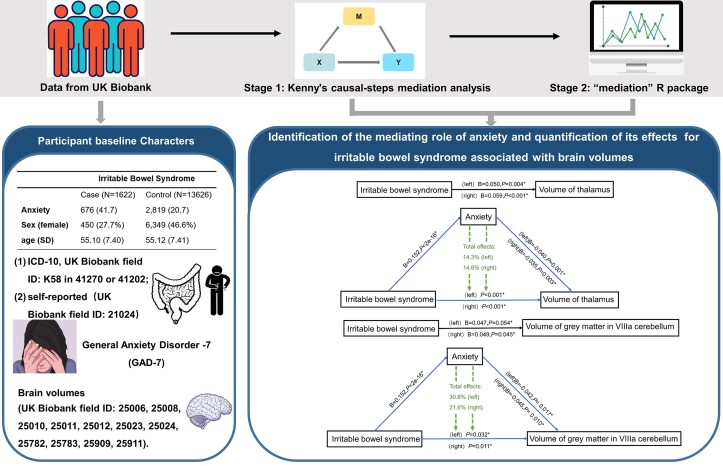

## Introduction

Irritable bowel syndrome (IBS) is a prevalent gastrointestinal syndrome characterized by bidirectional brain–gut axis disorders, affecting ∼7–21% of the global population.^1,2^ Although the exact mechanisms underlying symptom generation in IBS remain incompletely understood, a vital idea supports the interaction of psychological and social factors with physiological factors through the bidirectional communication between the central nervous system and the enteric nervous system.^3^ This may be caused primarily by alterations in the central nervous system (top–down model), or the gut (bottom–up model), or a combination of both.^4^ Considerable evidence has accumulated over the past few decades that multiple brain networks could mediate the effects of mood, affect and environmental factors on gut function and pain perception, causing visceral hypersensitivity and altered bowel habits.^5,6^ Activity in corticolimbic pontine networks mediates the effects of cognitions and emotions on the perception of discomfort and visceral pain.^5^ As expected, the results of functional neuroimaging findings in patients with IBS have so far been inconclusive, most commonly with increased regional activity in the insula and anterior midcingulate cortex.^7^ Patients with IBS also experienced an increase in hypothalamic grey matter compared with the healthy controls.^8^ In addition, IBS has been categorized as a ‘functional’ pain syndrome, and dysfunction of endogenous pain inhibition mechanisms is an attractive aetiological hypothesis in IBS, including the spino-bulbo-spinal feedback loop termed diffuse noxious inhibitory controls and the periaqueductal grey–rostroventral medulla network.^9^

Psychological stress is an important factor in the development of IBS. It is well known that IBS and the common mental disorders of anxiety often co-occur, and it is estimated that up to 60% of patients with IBS suffer from major psychosocial problems.^10^ Over the last decade, a growing number of research has demonstrated higher levels of anxiety in patients with IBS.^11^ For example, a clinic-based study from India suggested that the prevalence of anxiety in patients with IBS is 31.4%.^12^ In addition, non-pharmacological approaches and pharmacological strategies for stress-related alterations play a critical role in IBS management, such as antipsychotics and psychological intervention.^13,14^ Accumulative neuroimaging and volumetric studies in humans indicated that the specific involvement of the insular cortex and superior temporal areas is associated with anxiety disorders.^15^ Evidence from neuroimaging studies provided insight into the underlying association between brain volumes, IBS and anxiety from dysregulation of the brain–gut axis. In brief, they affect the central processing of visceral stimulation through the emotional circuit of the central nervous system, leading from the top–down to further amplify central pain.^16^ However, the interaction between anxiety symptoms, IBS and brain volume remains unclear.

In epidemiological studies, mediation analysis is often applied to assess the extent to which the effects of exposure can be explained by a certain set of hypothetical mediators. Mediation analysis investigates the mechanisms behind an observed relationship between an exposure variable and an outcome variable and examining how they relate to the mediator, to determine the total effect of the exposure on the outcome, the effect of the exposure that acts through a given set of mediators of interest (indirect effect) and the effect of the exposure unexplained by those same mediators (direct effect).^17,18^ When a mediator is a modifiable risk factor, this provides new opportunities for interventions to block the (partial) impact of exposure on outcomes.^19^ Anxiety, as the most common psychiatric disorder, has been shown to mediate associations among several phenotypes and diseases. Recent examples have addressed the mediating effect of anxiety on the association between cannabis use and attenuated positive psychotic symptoms,^20^ and the association between emotional reactivity and hypertension.^21^

In this study, based on the UK Biobank cohort, we detected the mediating role of anxiety on the associations between IBS and brain volumes by a two-stage mediation analysis. Briefly, we identified the mediating role of anxiety in the association between IBS and brain volumes in the first stage and further quantified the mediation effects of anxiety in the second stage. Sex stratification was conducted to investigate the role of sex in hypothesized mediation.

## Materials and methods

### UK Biobank cohort

As a large and population-based prospective study, UK Biobank (http://biobank.ndph.ox.ac.uk, application 46478) enrolled ∼500 000 participants across England, Wales and Scotland, aged 40–69 years in 2006–10. Extensive phenotypic and genotypic details were collected from participants, including physical measurements, sample analysis and longitudinal follow-up of health-related results.^22^ This study was conducted with the permission of UK Biobank and with access to the individuals’ health-related records. Ethical approval for the UK Biobank was granted by the National Health Service National Research Ethics Service (reference 11/NW/0382). The detailed characteristic descriptions of the study samples are summarized in Table 1.

**Table 1:** Characteristic of participants

	Irritable bowel syndrome	
	Case (*N* = 1622)	Control (*N* = 13 626)
Anxiety	676 (41.7)	2819 (20.7)
Sex (female)	450 (27.7%)	6349 (46.6%)
Age (SD)	55.10 (7.40)	55.12 (7.41)
Volume of grey matter (μm)	605 600.64 (52 934.55)	616 870.75 (54 503.06)
Volume of white matter (μm)	534 595.32 (58 057.65)	550 310.98 (61 274.00)
Volume of thalamus (left, μm)	7676.17 (761.47)	7781.81 (739.43)
Volume of thalamus (right, μm)	7488.62 (734.83)	7590.03 (719.63)
Volume of accumbens (left, μm)	497.83 (112.77)	502.68 (119.82)
Volume of accumbens (right, μm)	390.93 (107.72)	395.89 (110.06)
Volume of grey matter in the Frontal Pole (left, μm)	22 833.27 (2568.97)	23 228.54 (2700.08)
Volume of grey matter in the Frontal Pole (right, μm)	25 787.44 (2932.78)	26 226.09 (3015.00)
Volume of grey matter in the VIIIa cerebellum (left, μm)	3747.02 (688.53)	3756.61 (694.68)
Volume of grey matter in the VIIIa cerebellum (right, μm)	3808.96 (737.70)	3803.78 (742.56)

SD, standard deviation.

### Definitions of IBS and anxiety

Cases with IBS ascertained in the UK Biobank should meet the following two conditions: (i) the International Classification of Disease version 10 (ICD-10; UK Biobank field ID: K58 in 41270 or 41202); (ii) self-reported (UK Biobank field ID: 21024): answered ‘yes’ to the question ‘Have you ever been diagnosed with IBS?’. The healthy controls of IBS were defined according to a published article,^23^ and were excluded the individuals who self-reported IBS or were diagnosed as IBS according to ICD-10, the detailed control inclusion and exclusion criteria were presented in the Supplementary notes.

Brain volume–related indicators were measured by UK Biobank using MRI brain imaging including grey matter (UK Biobank code: 25006), white matter (UK Biobank code: 25008), grey and white matter (UK Biobank code: 25010), left and right thalamus (UK Biobank code: 25011:25012), left and right accumbens (UK Biobank code: 25023:25024), grey matter in the left frontal pole and grey matter in right frontal pole (UK Biobank code: 25782:25783) and grey matter in the left VIIIa cerebellum and grey matter in the right VIIIa cerebellum (UK Biobank code: 25909 and 25911). Before further analysis, brain volumes were normalized to 1 standard deviation.

The phenotype of anxiety is defined using the general anxiety disorder-7 (GAD-7). GAD-7 is a valid instrument of self-reported anxiety for subjects in both clinical and non-clinical settings,^24^ which is a 7-item anxiety scale with a total score (0–21) for screening and measuring anxiety severity by the 4-point ordinal scale.

### UK Biobank genotyping, imputation and quality control

A total of 488 377 participants were genotyped using either the Affymetrix UK BiLEVE Axiom Array or the Affymetrix UK Biobank Axiom arrays (Santa Clara, CA, USA).^25^ The imputation was carried out in chunks of ∼50 000 imputed markers with a 250 kb buffer region by IMPUTE4 (https://jmarchini.org/software/). Participants with inconsistencies between self-reported gender and genetic gender, without imputation data and ethical consent were excluded. Individuals were restricted to only ‘white British’ based on self-reported ethnicity (UK Biobank field ID: 21000). Genetic-related individuals were further excluded using the King software.^25^ Detailed descriptions of the array design, genotyping and quality control procedures are available elsewhere.^25^

### Statistical analysis

**Figure 1:**
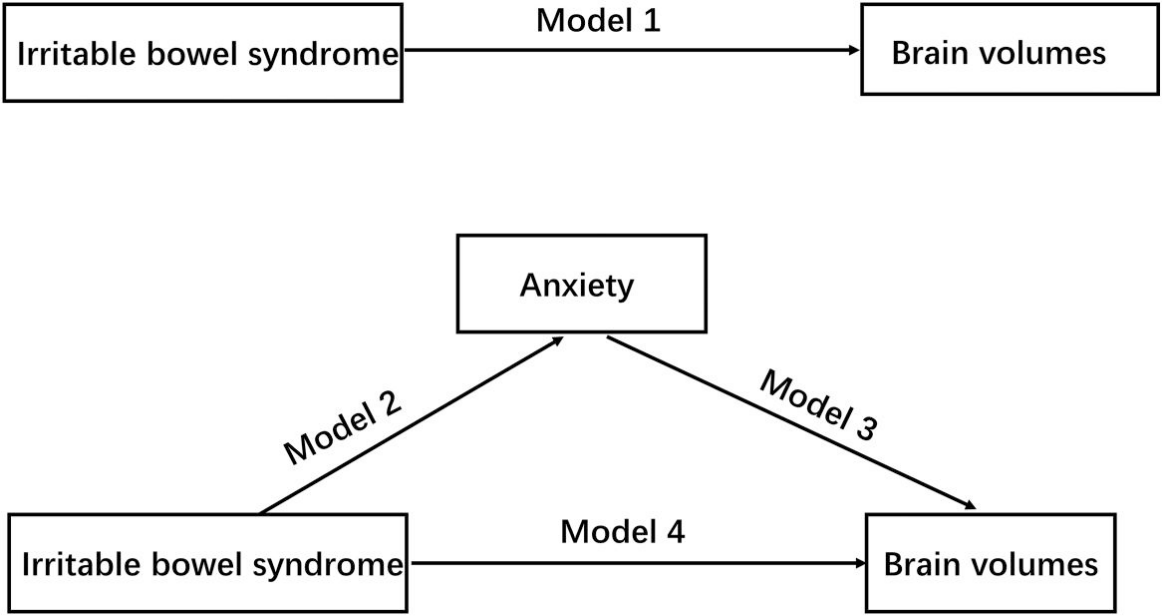
**The flow chart of the causal steps approach.** Mediation is tested through four regression models: Model 1: the association between IBS brain volumes; Model 2: the association between IBS and anxiety; Model 3: the association between anxiety and brain volumes; Model 4: the association between IBS and brain volumes with adjusted anxiety as covariates.

Mediation analysis was performed using a two-stage strategy to understand the observed association between brain volumes and IBS, which may be mediated by anxiety. In the first stage, according to the causal steps approach described by Baron and Kenny,^26^ we implemented four models for the exposure (X), outcome (Y) and mediator (M) separately to identify the candidate mediator using linear and logistic regression models: (i) IBS was significantly associated with brain volumes (Model 1: X∼Y), (ii) IBS was significantly association with anxiety (Model 2: X∼M) and (iii) anxiety was significantly related to brain volumes (Model 3: M∼Y). Anxiety would be considered as a candidate mediator only if all the three conditions were satisfied. Finally, (*i*) we detected the associations between IBS and brain volumes with adjusted anxiety as covariates (Model 4: X∼Y + M). The basic causal chain involved in mediation is given in Fig. 1.

In the second stage, we further quantified the effect of candidate mediators identified in the first stage through ‘mediation’ in the R package. We assessed the indirect effect [average causal mediation effect (ACME)] and direct effect [average direct effect (ADE)] based on the estimates derived from the first stage. The mediating function was used to evaluate the effect of anxiety on the association between IBS and brain volumes. The default number of simulations (1000) was used in this mediation analysis, which is the quasi-Bayesian Monte Carlo method based on normal approximation.^27^ Anxiety played a mediating role in the relationships between IBS and brain volumes only when the *P*-value of ACME results was statistically significant.

Briefly, the differences between the two mediation analyses can be concluded as follows: Firstly, the goal of the first stage is to evaluate the partial or complete mediation effect, while the second stage aims to obtain this indirect effect and see whether it is statistically significant. Secondly, the significance (confidence) level of these two mediation analyses is adjusted in different ways. Thirdly, the two mediation analyses apply to different conditions, where there is an exposure–mediator interaction. Fourthly, the inference of Bayesian mediation analysis (the second stage) does not depend on large-sample approximation, which is exact for small samples.^28^ Fifthly, the causal steps approach (the first stage) had the least statistical power compared with the quasi-Bayesian Monte Carlo approximation (the second stage) for significance testing.^29^

Sex, age, Townsend deprivation, alcohol use and 10 principle components of population structure were considered covariates. In addition, when brain volumes were considered as dependent variable, total intracranial volume was applied as a covariate in Models 3 and 4 of the first stage and in the mediator models of the second stage. The covariate of total intracranial volume included the sum of total grey matter (UK Biobank code: 25006), white matter (UK Biobank code: 25008) and cerebrospinal fluid (UK Biobank code: 25004). Data on brain volumes were standardized. All analyses were stratified by sex to assess sex-specific roles using R (version 3.5.1). *P* < 0.05 was considered as a significant threshold.

### Ethics approval statement

There is no ethical statement here, because all data were downloaded from the Internet.

## Results

### Identification of the mediating role of anxiety for IBS associated with brain volumes (the first stage)

In Model 1, 20 associations between IBS and brain volumes were detected, such as volume of thalamus (*P*_left_ = 3.59 × 10^−3^, *P*_right_ = 1.93 × 10^−2^), volume of grey matter in the VIIIa cerebellum (*P*_left_ = 5.46 × 10^−2^, *P*_right_ = 4.54 × 10^−2^) in the total sample, volume of thalamus (*P*_left_ = 1.93 × 10^−2^, *P*_right_ = 5.01 × 10^−3^) in males, and volume of grey matter in the VIIIa cerebellum (right) (*P* = 1.66 × 10^−2^) in females.

In Model 2, there were positive associations between IBS and anxiety in all populations (*β* = 0.15, *P* = 2 × 10^−16^), female samples (*β* = 0.16, *P* = 2 × 10^−16^) and male samples (*β* = 0.13, *P* = 2 × 10^−16^).

In Model 3, we observed two significant associations between anxiety and brain volumes in the total sample, including volume of thalamus [*P*_left_ = 9.75 × 10^−4^, *P*_right_ = 3.17 × 10^−3^; also significant in males (*P*_left_ = 6.58 × 10^−4^, *P*_right_ = 4.19 × 10^−3^)], and volume of grey matter in the VIIIa cerebellum [*P*_left_ = 1.68 × 10^−2^, *P*_right_ = 1.03 × 10^−2^; also significant in females (*P*_left_ = 3.77 × 10^−2^, *P*_right_ = 3.88 × 10^−3^)].

**Figure 2:**
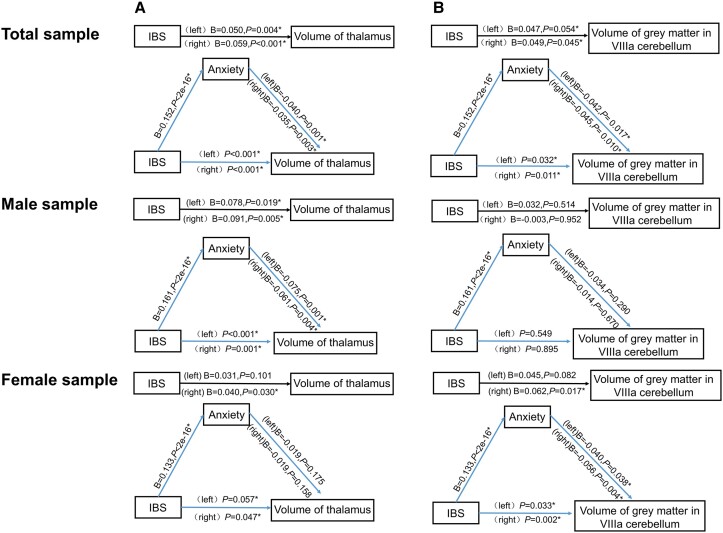
**Association between IBS and brain volumes via elevated anxiety through steps approach.** Based on Kenny’s causal steps mediation test, the associations between the IBS, anxiety and brain volumes were tested through the linear and logistic regression models, and phenotypic data of 15 248 participants from the UK Biobank were included in this study. (**A**) The mediating effects of anxiety in the association between IBS and volume of thalamus among total, male and female samples. (**B**) The mediating effects of anxiety in the association between IBS and volume of grey matter in VIIIa cerebellum in total, male and female samples. IBS, irritable bowel syndrome.

After adjusting anxiety as a covariate, we detected eight brain volumes that were associated with IBS. For example, volume of thalamus was associated with IBS in the total population (*P*_left_ = 1.16 × 10^−4^, *P*_right_ = 2.41 × 10^−4^) and male sample (*P*_left_ = 3.99 × 10^−4^, *P*_right_ = 1.20 × 10^−3^); volume of grey matter in the VIIIa cerebellum has associations with IBS in the total population (*P*_left_ = 3.22 × 10^−2^, *P*_right_ = 1.18 × 10^−2^) and the female sample (*P*_left_ = 3.39 × 10^−2^, *P*_right_ = 2.07 × 10^−3^), suggesting that anxiety regulates the association between IBS and certain brain volumes as a partial mediator. However, we investigated the mediator of anxiety in the relationship between IBS and 11 indicators related to brain volume, and found no complete mediation in these relationships. The detailed information is presented in Fig. 2 and Supplementary Fig. 1.

### Quantification of the mediation effects of anxiety on the association between IBS and brain volumes (the second stage)

Based on the mediated role of anxiety identified in the first stage, we further quantified the magnitude of the mediation effects by conducting parametric mediation analyses through the R package. Anxiety has significant indirect mediation effects for the association between IBS and volume of thalamus in the total sample (left: *β* = −0.008, *P* = 0.004; right: *β* = −0.007, *P* = 0.006) and the proportion of the total effect of IBS on volume of thalamus mediated by anxiety was 14.3% for left region and 14.6% for right region. Besides, anxiety indirectly mediates the associations between IBS and volume of grey matter in the VIIIa cerebellum (left: *β* = −0.013, *P* = 0.002; right: *β* = −0.010, *P* = 0.018), which indicated that anxiety mediates 30.8% for left region and 21.6% for the right region of the total effect of IBS on volume of grey matter in the VIIIa cerebellum. The same mediation effects of anxiety were also identified in the association between IBS and volume of grey matter in the VIIIa cerebellum (left: *β* = −0.012, *P* = 0.012, right: *β* = −0.012, *P* = 0.010) in females, the association between IBS and volume of thalamus (left: *β* = −0.010, *P* = 0.020; right: *β* = −0.010, *P* = 0.018) in males (Table 2 and Supplementary Table 1).

**Table 2:** Direct and indirect associations of irritable bowel syndrome with brain volumes mediated via anxiety

		Total sample				Female sample				Male sample			
		B	95% CI Lower	95% CI upper	*P*-value	B	95% CI Lower	95% CI upper	*P*-value	B	95% CI Lower	95% CI upper	*P*-value
Volume of thalamus (left)	ACME	−0.008	−0.014	0.000	0.004	−0.005	−0.012	0.000	0.110	−0.010	−0.019	0.000	0.020
	ADE	0.062	0.024	0.100	<2e-16	0.046	0.008	0.090	0.026	0.085	0.013	0.160	0.016
	Total effect	0.055	0.016	0.090	<2e-16	0.041	0.004	0.080	0.036	0.075	0.005	0.150	0.036
	PM, %	14.3	−0.495	−0.030	0.004	12.8	−0.768	0.090	0.142	12.8	−0.735	0.020	0.056
Volume of thalamus (right)	ACME	−0.007	−0.013	0.000	0.006	−0.005	−0.011	0.000	0.130	−0.010	−0.019	0.000	0.018
	ADE	0.058	0.020	0.090	0.002	0.036	−0.004	0.080	0.080	0.095	0.024	0.160	0.006
	Total effect	0.051	0.014	0.090	0.008	0.031	−0.009	0.070	0.110	0.085	0.016	0.150	0.014
	PM, %	14.6	−0.508	−0.030	0.014	13.8	−1.542	0.840	0.230	11.4	−0.504	−0.010	0.032
Volume of grey matter in the VIIIa cerebellum (left)	ACME	−0.013	−0.021	−0.010	0.002	−0.012	−0.022	0.000	0.012	−0.011	−0.025	0.000	0.096
	ADE	0.050	−0.003	0.100	0.068	0.056	0.000	0.110	0.052	−0.002	−0.109	0.100	0.992
	Total effect	0.036	−0.016	0.090	0.172	0.043	−0.011	0.100	0.122	−0.013	−0.121	0.090	0.832
	PM, %	30.8	−3.603	2.420	0.174	25.9	−2.468	1.450	0.134	8.6	−2.587	4.540	0.836
Volume of grey matter in the VIIIa cerebellum (right)	ACME	−0.010	−0.018	0.000	0.018	−0.012	−0.021	0.000	0.010	−0.005	−0.019	0.010	0.490
	ADE	0.047	−0.010	0.100	0.100	0.065	0.008	0.120	0.014	−0.030	−0.138	0.080	0.590
	Total effect	0.037	−0.023	0.090	0.192	0.054	−0.002	0.110	0.070	−0.034	−0.143	0.080	0.540
	PM, %	21.6	−2.375	2.360	0.210	20.3	−1.761	1.380	0.080	3.9	−1.950	1.420	0.800

ACME, average causal mediation effect; ADE, average direct effect; PM, proportion mediated.

## Discussion

Previous studies proposed that altered central processing of visceral stimuli in IBS is at least partly mediated by symptoms of anxiety.^30^ This study applied a two-stage strategy to explore the exact mediating role of anxiety in the correlation between brain volumes and IBS. Specifically, we first performed an observational association between IBS and brain volumes, IBS and anxiety, IBS and brain volumes and identified the candidate role of anxiety in mediating the association between IBS and brain volumes in various regions. We then quantified the statistical significance of mediation effect estimates by decomposing the total effect into ACME and ADE, further confirmed the indicated mediation effects of anxiety.

As a relay station for every sensory input to the cerebral cortex, thalamus has extensive functional connectivity with cortical and other subcortical regions.^31^ Recent brain imaging studies have revealed the long-term microstructural changes within the brain in patients with chronically recurring visceral pain, particularly in regions associated with the integration of sensory information and corticothalamic modulation,^32^ which are consistent with our results. By comparing the structural magnetic resonance data from patients with IBS and demographically similar healthy subjects, Mao *et al*.^33^ indicated that patients with IBS showed significantly larger normalized volumes on the right thalamus than healthy controls, which may be partly attributable to the increased engagement in regions associated with emotional arousal and endogenous pain modulation.^34^ The most common symptom of IBS is chronically recurring abdominal pain. The thalamus is involved in the introduction of visceral and pain-related information from throughout the body in patients with IBS, first routed to the thalamus and then reached to the insula and anterior cingulate cortex, respectively.^35^ The biopsychological model of IBS showed that functional abdominal pain secondarily influences mental health.^36^ There is a strong correlation between the development of IBS and psychological or psychosocial stressors, especially higher levels of anxiety.^37^ In this study, anxiety was identified as an indirect mediator in the association between IBS and volume of thalamus in the total and male samples. Functional MRI studies showed abnormal spontaneous activities in brain regions related to pain processing, such as thalamus and insula and emotion regulation in patients with IBS and partly due to anxiety.^38,39^ Wang *et al*.^40^ also observed higher anxiety scores in patients with IBS and gradual increases in thalamus with uncomfortable rectal distension, elucidating the role of psychological factors in the pathogenesis of IBS. Anxiety explains the abnormal grey matter density in the anterior/medial thalamus in patients with IBS.^41,42^

Symptoms of anxiety are frequently seen in chronic pain disorders such as IBS, and they regulate the activation of visceral sensory signals in the thalamus and cerebellum.^43^ The cerebellum contributes to the neural processing of both emotions and painful stimuli.^44^ There was a greater cerebellar activity during the acquisition of conditioned pain-related fear in patients with IBS.^45^ Meanwhile, a voxel-based meta-analysis demonstrated a significant increase in brain activities in the left cerebellum in patients with IBS to facilitate the regulation of signals from higher cortical areas to process emotion and anxiety.^46^ These abnormal activities may reflect the modulatory role of anxiety in the regulation of gastrointestinal pain and in patients with IBS, which is consistent with our results that IBS is positively related to the volume of grey matter in the VIIIa cerebellum partly regulated by anxiety. Notably, different regions within the cerebellum were reported to take part in the processing of acute and chronic pain; for example, regions involved in sensory-motor processing, like VIIIa, would be activated by heat pain.^47^ Likewise, L. VIII in the posterior lobe is involved in sensorimotor processing, which is related to the sensory dimensions of pain, and suggests the strongest correlation with the processing of non-painful visceral stimuli.^43^

IBS is known to have a higher incidence in women associated with mental disorders. In this study, there was also a gender difference in the mediating role of anxiety. Anxiety regulated the association between IBS and volume of thalamus only in men and between IBS and volume of grey matter in the VIIIa cerebellum only in women. Berman *et al*.^48^ pointed out that rectal pressure increased regional cerebral blood flow in areas commonly associated with somatic pain, including thalamus, and activated the area much more strongly in men with IBS. Furthermore, the visceral pain studies showed increased cerebellum activations occurring mostly in female patients with IBS.^49^

There are several limitations to the present study. First of all, IBS is classified into diarrhoea type (IBS-D), constipation type (IBS-C), mixed type and undefined type according to the predominant stool pattern, which was not considered in this study. Secondly, despite growing evidence of activation of visceral sensory signals within the cerebellum, linking the functions of pain processes to the development of IBS, previous studies failed to explore the function of grey matter in the VIIIa cerebellum, which limited an adequate explanation of our results. Thirdly, due to the fact that the data applied in our analysis were extracted from the UK Biobank and were almost exclusively for the elderly European population, caution needs to be exercised when applying our results to other races.

## Conclusion

In this study, we found that IBS was positively associated with the volumes of thalamus and grey matter in the VIIIa cerebellum, which may be partly mediated by anxiety. This study revealed the indirect mediating role of anxiety in the association between IBS and brain volumes and promoted our understanding of the functional mechanisms of IBS and its related psychosocial factors.

## Supplementary Material

fcad116_Supplementary_Data

## Data Availability

The UK Biobank data are available through the UK Biobank Access Management System https://www.ukbiobank.ac.uk/. The authors will return the derived data fields following UK Biobank policy; in due course, they will be available through the UK Biobank Access Management System.

## Abbreviations

ACME =: average causal mediation effect
ADE =: average direct effect
GAD-7 =: general anxiety disorder-7
IBS =: irritable bowel syndrome
ICD-10 =: International Classification of Disease version 10

## References

[1] Longstreth GF , Thompson WG, Chey WD, Houghton LA, Mearin F, Spiller RC. Functional bowel disorders. Gastroenterology. 2006;130(5):1480–1491. 16678561 10.1053/j.gastro.2005.11.061

[2] Lovell RM , Ford AC. Global prevalence of and risk factors for irritable bowel syndrome: A meta-analysis. Clin Gastroenterol Hepatol. 2012;10(7):712–721.e4. 22426087 10.1016/j.cgh.2012.02.029

[3] Surdea-Blaga T , Băban A, Dumitrascu DL. Psychosocial determinants of irritable bowel syndrome. World J Gastroenterol. 2012;18(7):616–626. 22363132 10.3748/wjg.v18.i7.616 PMC3281218

[4] Stasi C , Rosselli M, Bellini M, Laffi G, Milani S. Altered neuro-endocrine-immune pathways in the irritable bowel syndrome: The top-down and the bottom-up model. J Gastroenterol. 2012;47(11):1177–1785. 22766747 10.1007/s00535-012-0627-7

[5] Mayer EA , Tillisch K. The brain-gut axis in abdominal pain syndromes. Annu Rev Med. 2011;62:381–396. 21090962 10.1146/annurev-med-012309-103958 PMC3817711

[6] Bhatt RR , Gupta A, Labus JS, et al Altered brain structure and functional connectivity and its relation to pain perception in girls with irritable bowel syndrome. Psychosom Med. 2019;81(2):146–154. 30615602 10.1097/PSY.0000000000000655 PMC6355369

[7] Labus JS , Vianna EP, Tillisch K, Naliboff B, Mayer EA. Brain response during pelvic visceral distension in healthy controls and patients with irritable bowel syndrome. Neurogastroenterol Motil. 2009;21(Suppl 1):80.

[8] Blankstein U , Chen J, Diamant NE, Davis KD. Altered brain structure in irritable bowel syndrome: Potential contributions of pre-existing and disease-driven factors. Gastroenterology. 2010;138(5):1783–1789. 20045701 10.1053/j.gastro.2009.12.043

[9] Gebhart GF . Descending modulation of pain. Neurosci Biobehav Rev. 2004;27(8):729–737. 15019423 10.1016/j.neubiorev.2003.11.008

[10] Levy RL , Olden KW, Naliboff BD, et al Psychosocial aspects of the functional gastrointestinal disorders. Gastroenterology. 2006;130(5):1447–1458. 16678558 10.1053/j.gastro.2005.11.057

[11] Fond G , Loundou A, Hamdani N, et al Anxiety and depression comorbidities in irritable bowel syndrome (IBS): A systematic review and meta-analysis. Eur Arch Psychiatry Clin Neurosci. 2014;264(8):651–660. 24705634 10.1007/s00406-014-0502-z

[12] Kabra N , Nadkarni A. Prevalence of depression and anxiety in irritable bowel syndrome: A clinic based study from India. Indian J Psychiatry. 2013;55(1):77–80. 23439939 10.4103/0019-5545.105520 PMC3574461

[13] Qin HY , Cheng CW, Tang XD, Bian ZX. Impact of psychological stress on irritable bowel syndrome. World J Gastroenterol. 2014;20(39):14126–14131. 25339801 10.3748/wjg.v20.i39.14126 PMC4202343

[14] Graff LA , Clara I, Walker JR, et al Changes in fatigue over 2 years are associated with activity of inflammatory bowel disease and psychological factors. Clin Gastroenterol Hepatol. 2013;11(9):1140–1146. 23602816 10.1016/j.cgh.2013.03.031

[15] van Tol MJ , van der Wee NJ, van den Heuvel OA, et al Regional brain volume in depression and anxiety disorders. Arch Gen Psychiatry. 2010;67(10):1002–1011. 20921116 10.1001/archgenpsychiatry.2010.121

[16] Mudyanadzo TA , Hauzaree C, Yerokhina O, Architha NN, Ashqar HM. Irritable bowel syndrome and depression: A shared pathogenesis. Cureus. 2018;10(8):e3178. 30357038 10.7759/cureus.3178 PMC6197537

[17] Richiardi L , Bellocco R, Zugna D. Mediation analysis in epidemiology: Methods, interpretation and bias. Int J Epidemiol. 2013;42(5):1511–1519. 24019424 10.1093/ije/dyt127

[18] Valeri L , Vanderweele TJ. Mediation analysis allowing for exposure-mediator interactions and causal interpretation: Theoretical assumptions and implementation with SAS and SPSS macros. Psychol Methods. 2013;18(2):137–150. 23379553 10.1037/a0031034 PMC3659198

[19] Groeniger J O , Burdorf A. Advancing mediation analysis in occupational health research. Scand J Work Environ Health. 2020;46(2):113–116. 31950195 10.5271/sjweh.3886

[20] Reeves LE , Anglin DM, Heimberg RG, et al Anxiety mediates the association between cannabis use and attenuated positive psychotic symptoms. Psychiatry Res. 2014;218(1-2):180–186. 24745470 10.1016/j.psychres.2014.03.040

[21] Ifeagwazi CM , Egberi HE, Chukwuorji JC. Emotional reactivity and blood pressure elevations: Anxiety as a mediator. Psychol Health Med. 2018;23(5):585–592. 29105504 10.1080/13548506.2017.1400670

[22] Sudlow C , Gallacher J, Allen N, et al UK Biobank: An open access resource for identifying the causes of a wide range of complex diseases of middle and old age. PLoS Med. 2015;12(3):e1001779. 10.1371/journal.pmed.1001779 PMC4380465 25826379

[23] Eijsbouts C , Zheng T, Kennedy NA, Bonfiglio F, Anderson CA. Genome-wide analysis of 53,400 people with irritable bowel syndrome highlights shared genetic pathways with mood and anxiety disorders. Nat Genet. 2021;53(11):1543–1552. 34741163 10.1038/s41588-021-00950-8 PMC8571093

[24] Spitzer RL , Kroenke K, Williams JB, Löwe B. A brief measure for assessing generalized anxiety disorder: The GAD-7. Arch Intern Med. 2006;166(10):1092–1097. 16717171 10.1001/archinte.166.10.1092

[25] Bycroft C , Freeman C, Petkova D, et al The UK Biobank resource with deep phenotyping and genomic data. Nature. 2018;562(7726):203–209. 30305743 10.1038/s41586-018-0579-z PMC6786975

[26] Baron RM , Kenny DA. The moderator-mediator variable distinction in social psychological research: Conceptual, strategic, and statistical considerations. J Pers Soc Psychol. 1986;51(6):1173–1182. 3806354 10.1037//0022-3514.51.6.1173

[27] Imai K , Keele L, Tingley D. A general approach to causal mediation analysis. Psychol Methods. 2010;15(4):309–334. 20954780 10.1037/a0020761

[28] Yuan Y , MacKinnon DP. Bayesian mediation analysis. Psychol Methods. 2009;14(4):301–322. 19968395 10.1037/a0016972 PMC2885293

[29] MacKinnon DP , Lockwood CM, Hoffman JM, West SG, Sheets V. A comparison of methods to test mediation and other intervening variable effects. Psychol Methods. 2002;7(1):83–104. 11928892 10.1037/1082-989x.7.1.83 PMC2819363

[30] Elsenbruch S , Rosenberger C, Enck P, Forsting M, Schedlowski M, Gizewski ER. Affective disturbances modulate the neural processing of visceral pain stimuli in irritable bowel syndrome: An fMRI study. Gut. 2010;59(4):489–495. 19651629 10.1136/gut.2008.175000

[31] Mastropasqua C , Bozzali M, Spanò B, Koch G, Cercignani M. Functional anatomy of the thalamus as a model of integrated structural and functional connectivity of the human brain in vivo. Brain Topogr. 2015;28(4):548–558. 25549779 10.1007/s10548-014-0422-2

[32] Ellingson BM , Mayer E, Harris RJ, et al Diffusion tensor imaging detects microstructural reorganization in the brain associated with chronic irritable bowel syndrome. Pain. 2013;154(9):1528–1541. 23721972 10.1016/j.pain.2013.04.010 PMC3758125

[33] Mao CP , Chen FR, Sun HH, et al Larger regional volume of the thalamus in diarrhea-predominant irritable bowel syndrome: A cross-sectional study. Brain Imaging Behav. 2020;14(6):2302–2310. 31468373 10.1007/s11682-019-00181-w

[34] Tillisch K , Mayer EA, Labus JS. Quantitative meta-analysis identifies brain regions activated during rectal distension in irritable bowel syndrome. Gastroenterology. 2011;140(1):91–100. 20696168 10.1053/j.gastro.2010.07.053 PMC3253553

[35] Mayer EA , Aziz Q, Coen S, et al Brain imaging approaches to the study of functional GI disorders: A Rome working team report. Neurogastroenterol Motil. 2009;21(6):579–596. 19646070 10.1111/j.1365-2982.2009.01304.x PMC3829384

[36] Walter SA , Jones MP, Talley NJ, et al Abdominal pain is associated with anxiety and depression scores in a sample of the general adult population with no signs of organic gastrointestinal disease. Neurogastroenterol Motil. 2013;25(9):741-e576. 10.1111/nmo.12155 23692044

[37] Sugaya N , Nomura S, Shimada H. Relationship between cognitive factors and anxiety in individuals with irritable bowel syndrome. Int J Behav Med. 2012;19(3):308–315. 21935740 10.1007/s12529-011-9195-0

[38] Ma X , Li S, Tian J, et al Altered brain spontaneous activity and connectivity network in irritable bowel syndrome patients: A resting-state fMRI study. Clin Neurophysiol. 2015;126(6):1190–1197. 25454279 10.1016/j.clinph.2014.10.004

[39] Qi R , Liu C, Ke J, et al Intrinsic brain abnormalities in irritable bowel syndrome and effect of anxiety and depression. Brain Imaging Behav. 2016;10(4):1127–1134. 26556814 10.1007/s11682-015-9478-1

[40] Wang D , Zhang X, Zhang X, Huang Z, Song Y. Magnetic resonance imaging analysis of brain function in patients with irritable bowel syndrome. BMC Gastroenterol. 2017;17(1):148. 29216847 10.1186/s12876-017-0673-y PMC5721622

[41] Davis KD , Pope G, Chen J, Kwan CL, Crawley AP, Diamant NE. Cortical thinning in IBS: Implications for homeostatic, attention, and pain processing. Neurology. 2008;70(2):153–154. 17959767 10.1212/01.wnl.0000295509.30630.10

[42] Seminowicz DA , Labus JS, Bueller JA, et al Regional gray matter density changes in brains of patients with irritable bowel syndrome. Gastroenterology. 2010;139(1):48–57.e2. 20347816 10.1053/j.gastro.2010.03.049 PMC2902717

[43] Rosenberger C , Thürling M, Forsting M, Elsenbruch S, Timmann D, Gizewski ER. Contributions of the cerebellum to disturbed central processing of visceral stimuli in irritable bowel syndrome. Cerebellum. 2013;12(2):194–198. 22910984 10.1007/s12311-012-0413-3

[44] Weintraub D , Aarsland D, Chaudhuri KR, et al The neuropsychiatry of Parkinson’s disease: Advances and challenges. Lancet Neurol. 2022;21(1):89–102. 34942142 10.1016/S1474-4422(21)00330-6 PMC8800169

[45] Claassen J , Labrenz F, Ernst TM, et al Altered cerebellar activity in visceral pain-related fear conditioning in irritable bowel syndrome. Cerebellum. 2017;16(2):508–517. 27797090 10.1007/s12311-016-0832-7

[46] Su C , Liu W, Wang Q, et al Abnormal resting-state local spontaneous functional activity in irritable bowel syndrome patients: A meta-analysis. J Affect Disord. 2022;302:177–184. 35066011 10.1016/j.jad.2022.01.075

[47] Borsook D , Moulton EA, Tully S, Schmahmann JD, Becerra L. Human cerebellar responses to brush and heat stimuli in healthy and neuropathic pain subjects. Cerebellum. 2008;7(3):252–272. 18418691 10.1007/s12311-008-0011-6

[48] Berman S , Munakata J, Naliboff BD, et al Gender differences in regional brain response to visceral pressure in IBS patients. Eur J Pain. 2000;4(2):157–172. 10957697 10.1053/eujp.2000.0167

[49] Guleria A , Karyampudi A, Singh R, et al Mapping of brain activations to rectal balloon distension stimuli in male patients with irritable bowel syndrome using functional magnetic resonance imaging. J Neurogastroenterol Motil. 2017;23(3):415–427. 28192648 10.5056/jnm16148 PMC5503292

