# Supplementary material for: Evaluating the role of anxiety on the association between irritable bowel syndrome and brain volumes: a mediation analysis in the UK Biobank cohort
Source: Brain Commun. 2023 Apr 11;5(2):fcad116. doi: 10.1093/braincomms/fcad116 (PMC10116581; doi:10.1093/braincomms/fcad116)
Supplement: fcad116_Supplementary_Data [file fcad116_supplementary_data.docx]

**Supplementary notes**

In the UK Biobank Discovery Cohort meta-analysis for IBS, two control panels were identified based on the Digestive Health Questionnaire (DHQ). DHQ response status (responder or nonresponder) was matched to the respective case panel. The same exclusion was performed for both - namely exclusions as per the cases, but in addition, patients who were coded as having diverticular disease, dyspepsia, infectious gastroenteritis, or gallbladder surgery were excluded, because IBS often overlaps or is misspecified in these diagnostic categories, as well as IBS itself. DHQ-responder controls were known to have no significant abdominal symptoms: they reported abdominal pain on less than 1 day per month; hard/lumpy or loose/watery stools were "never" or at most "sometimes" in the past three months. For DHQ nonresponder controls, we lacked this phenotypic information and excluded as described above [1].

**Supplementary Table 1. Direct and Indirect Associations of Irritable Bowel Syndrome with brain volumes Mediated via Anxiety**

|  | |  | **Total sample** | | | | **Female sample** | | | | **Male sample** | | | |
| --- | --- | --- | --- | --- | --- | --- | --- | --- | --- | --- | --- | --- | --- | --- |
|  | |  | B | 95%CI Lower | 95%CI upper | *P* | B | 95%CI Lower | 95%CI upper | *P* | B | 95%CI Lower | 95%CI upper | *P* |
| **Volume of grey matter** | ACME | | -0.002 | -0.005 | 0.000 | 0.100 | -0.002 | -0.006 | 0.000 | 0.154 | -0.002 | -0.007 | 0.000 | 0.276 |
|  | ADE | | 0.009 | -0.012 | 0.030 | 0.390 | 0.028 | 0.005 | 0.050 | 0.020 | -0.035 | -0.074 | 0.000 | 0.076 |
|  | Total effect | | 0.007 | -0.014 | 0.030 | 0.550 | 0.026 | 0.003 | 0.050 | 0.032 | -0.038 | -0.076 | 0.000 | 0.054 |
|  | PM, % | | 13.9 | -2.359 | 4.790 | 0.600 | 8.9 | -0.544 | 0.050 | 0.170 | 6.100 | -0.130 | 0.500 | 0.306 |
| **Volume of white matter** | ACME | | 0.002 | -0.001 | 0.000 | 0.180 | 0.000 | -0.003 | 0.000 | 0.980 | 0.004 | 0.000 | 0.010 | 0.044 |
|  | ADE | | 0.013 | -0.005 | 0.030 | 0.136 | -0.002 | -0.022 | 0.020 | 0.830 | 0.051 | 0.017 | 0.080 | 0.004 |
|  | Total effect | | 0.015 | -0.002 | 0.030 | 0.092 | -0.002 | -0.022 | 0.020 | 0.850 | 5.53e-02 | 2.23e-02 | 0.090 | <2e-16 |
|  | PM, % | | 10.7 | -0.597 | 0.910 | 0.264 | 0.004 | -2.092 | 1.610 | 0.980 | 7.6 | 0.002 | 0.270 | 0.044 |
| **Volume of thalamus (left)** | ACME | | -0.008 | -0.014 | 0.000 | 0.004 | -0.005 | -0.012 | 0.000 | 0.110 | -0.010 | -0.019 | 0.000 | 0.020 |
|  | ADE | | 0.062 | 0.024 | 0.100 | <2e-16 | 0.046 | 0.008 | 0.090 | 0.026 | 0.085 | 0.013 | 0.160 | 0.016 |
|  | Total effect | | 0.055 | 0.016 | 0.090 | <2e-16 | 0.041 | 0.004 | 0.080 | 0.036 | 0.075 | 0.005 | 0.150 | 0.036 |
|  | PM, % | | 14.3 | -0.495 | -0.030 | 0.004 | 12.8 | -0.768 | 0.090 | 0.142 | 12.8 | -0.735 | 0.020 | 0.056 |
| **Volume of thalamus (right)** | ACME | | -0.007 | -0.013 | 0.000 | 0.006 | -0.005 | -0.011 | 0.000 | 0.130 | -0.010 | -0.019 | 0.000 | 0.018 |
|  | ADE | | 0.058 | 0.020 | 0.090 | 0.002 | 0.036 | -0.004 | 0.080 | 0.080 | 0.095 | 0.024 | 0.160 | 0.006 |
|  | Total effect | | 0.051 | 0.014 | 0.090 | 0.008 | 0.031 | -0.009 | 0.070 | 0.110 | 0.085 | 0.016 | 0.150 | 0.014 |
|  | PM, % | | 14.6 | -0.508 | -0.030 | 0.014 | 13.8 | -1.542 | 0.840 | 0.230 | 11.4 | -0.504 | -0.010 | 0.032 |
| **Volume of accumbens (left)** | ACME | | -0.007 | -0.015 | 0.000 | 0.066 | -0.008 | -0.018 | 0.000 | 0.100 | -0.004 | -0.017 | 0.010 | 0.470 |
|  | ADE | | 0.027 | -0.025 | 0.080 | 0.324 | 0.019 | -0.042 | 0.080 | 0.530 | 0.029 | -0.074 | 0.130 | 0.560 |
|  | Total effect | | 0.020 | -0.031 | 0.070 | 0.444 | 0.011 | -0.050 | 0.080 | 0.730 | 0.025 | -0.075 | 0.120 | 0.610 |
|  | PM, % | | 18.8 | -3.312 | 2.570 | 0.470 | 12.6 | -4.513 | 5.220 | 0.760 | 4.5 | -2.892 | 1.440 | 0.760 |
| **Volume of accumbens (right)** | ACME | | -0.005 | -0.013 | 0.000 | 0.180 | -0.010 | -0.019 | 0.000 | 0.044 | 0.003 | -0.010 | 0.020 | 0.650 |
|  | ADE | | 0.023 | -0.029 | 0.080 | 0.420 | 0.005 | -0.058 | 0.070 | 0.874 | 0.049 | -0.048 | 0.150 | 0.330 |
|  | Total effect | | 0.018 | -0.032 | 0.070 | 0.520 | -0.004 | -0.068 | 0.060 | 0.890 | 0.052 | -0.044 | 0.150 | 0.310 |
|  | PM, % | | 11.2 | -2.073 | 2.560 | 0.610 | 10.500 | -5.069 | 3.690 | 0.870 | 3.500 | -0.864 | 0.930 | 0.720 |
| **Volume of grey matter in Frontal Pole (left)** | ACME | | -0.003 | -0.008 | 0.000 | 0.310 | -0.004 | -0.011 | 0.000 | 0.280 | -0.002 | -0.010 | 0.010 | 0.710 |
|  | ADE | | 0.038 | 0.002 | 0.080 | 0.034 | 0.077 | 0.036 | 0.120 | <2e-16 | -0.042 | -0.108 | 0.030 | 0.230 |
|  | Total effect | | 0.035 | -0.001 | 0.070 | 0.060 | 0.073 | 0.032 | 0.110 | <2e-16 | -0.043 | -0.111 | 0.030 | 0.210 |
|  | PM, % | | 7.7 | -0.690 | 0.230 | 0.350 | 5.0 | -0.224 | 0.040 | 0.280 | 2.4 | -0.759 | 0.710 | 0.790 |
| **Volume of grey matter in Frontal Pole (right)** | ACME | | -0.002 | -0.007 | 0.000 | 0.462 | -0.006 | -0.013 | 0.000 | 0.094 | 0.003 | -0.005 | 0.010 | 0.410 |
|  | ADE | | 0.032 | -0.005 | 0.070 | 0.086 | 0.056 | 0.013 | 0.100 | 0.012 | -0.013 | -0.080 | 0.060 | 0.730 |
|  | Total effect | | 0.030 | -0.006 | 0.060 | 0.092 | 0.050 | 0.008 | 0.090 | 0.024 | -0.009 | -0.075 | 0.060 | 0.790 |
|  | PM, % | | 5.7 | -0.792 | 0.320 | 0.530 | 11.8 | -0.617 | 0.040 | 0.114 | 3.2 | -2.186 | 1.540 | 0.850 |
| **Volume of grey matter in VIIIa cerebellum (left)** | ACME | | -0.013 | -0.021 | -0.010 | 0.002 | -0.012 | -0.022 | 0.000 | 0.012 | -0.011 | -0.025 | 0.000 | 0.096 |
|  | ADE | | 0.050 | -0.003 | 0.100 | 0.068 | 0.056 | 0.000 | 0.110 | 0.052 | -0.002 | -0.109 | 0.100 | 0.992 |
|  | Total effect | | 0.036 | -0.016 | 0.090 | 0.172 | 0.043 | -0.01 | 0.100 | 0.122 | -0.013 | -0.121 | 0.090 | 0.832 |
|  | PM, % | | 30.8 | -3.603 | 2.420 | 0.174 | 25.9 | -2.468 | 1.450 | 0.134 | 8.6 | -2.587 | 4.540 | 0.836 |
| **Volume of grey matter in VIIIa cerebellum (right)** | ACME | | -0.010 | -0.018 | 0.000 | 0.018 | -0.012 | -0.021 | 0.000 | 0.010 | -0.005 | -0.019 | 0.010 | 0.490 |
|  | ADE | | 0.047 | -0.010 | 0.100 | 0.100 | 0.065 | 0.008 | 0.120 | 0.014 | -0.030 | -0.138 | 0.080 | 0.590 |
|  | Total effect | | 0.037 | -0.023 | 0.090 | 0.192 | 0.054 | -0.002 | 0.110 | 0.070 | -0.034 | -0.143 | 0.080 | 0.540 |
|  | PM, % | | 21.6 | -2.375 | 2.360 | 0.210 | 20.3 | -1.761 | 1.380 | 0.080 | 3.9 | -1.950 | 1.420 | 0.800 |
| **Volume of gray and white matter** | ACME | | 0.000 | -0.001 | 0.000 | 0.720 | -0.001 | -0.002 | 0.000 | 0.068 | 0.001 | -0.001 | 0.000 | 0.170 |
|  | ADE | | 0.012 | 0.005 | 0.020 | <2e-16 | 0.013 | 0.005 | 0.020 | 0.002 | 0.011 | -0.004 | 0.020 | 0.190 |
|  | Total effect | | 0.012 | 0.005 | 0.020 | <2e-16 | 0.011 | 0.004 | 0.020 | 0.002 | 0.012 | -0.002 | 0.030 | 0.120 |
|  | PM, % | | 1.8 | -0.152 | 0.090 | 0.720 | 10.0 | -0.334 | 0.010 | 0.070 | 8.9 | -0.634 | 1.220 | 0.260 |

Abbreviations: ACME, average causal mediation effect; ADE, average direct effect; PM, proportion mediated.

**Supplementary Figure 1. Association between IBS and brain volumes via elevated anxiety through steps approach**


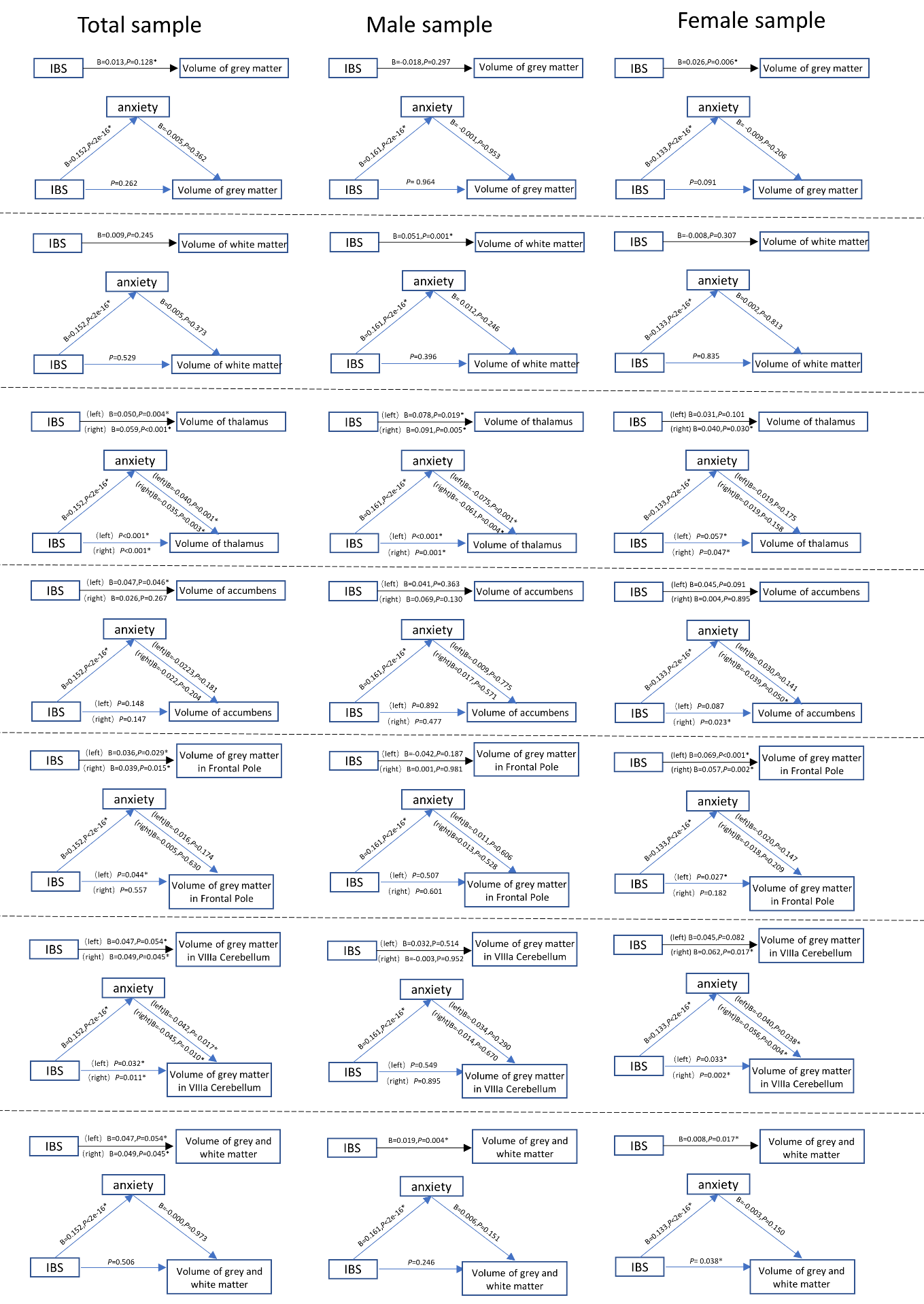
Note: Based on the Kenny’s causal-steps mediation test, the associations between the IBS, anxiety, and brain volumes were tested through the linear and logistic regression models, and phenotypic data of 15,248 participants from UK Biobank were included in this study.

Abbreviations: IBS, Irritable Bowel Syndrome.

**Supplementary Reference**

1. Eijsbouts, C., et al., *Genome-wide analysis of 53,400 people with irritable bowel syndrome highlights shared genetic pathways with mood and anxiety disorders.* Nat Genet, 2021. **53**(11): p. 1543-1552.
